# Joint linkage and imprinting analyses of GAW15 rheumatoid arthritis and gene expression data

**DOI:** 10.1186/1753-6561-1-s1-s53

**Published:** 2007-12-18

**Authors:** Xiaojun Zhou, Wei Chen, Michael D Swartz, Yue Lu, Robert Yu, Christopher I Amos, Chih-Chieh Wu, Sanjay Shete

**Affiliations:** 1 Department of Epidemiology, Unit 1340, The University of Texas M. D. Anderson Cancer Center, 1155 Hermann Pressler Boulevard, Houston, TX, 77030, USA

## Abstract

**Background:**

Genomic imprinting is a mechanism in which the expression of a gene copy depends upon the sex of the parent from which it was inherited. This mechanism is now well recognized in humans, and the deregulation of imprinted genes has been implicated in a number of diseases. In this study, we performed a genome-wide joint linkage and imprinting scan using two data sets provided by Genetic Analysis Workshop 15 (GAW15).

**Results:**

The first data set was high-risk rheumatoid arthritis families collected by the North American Rheumatoid Arthritis Consortium. We used both model-based and model-free methods of joint linkage and imprinting analyses. Although a genome scan of rheumatoid arthritis families using GENEHUNTER-MODSCORE suggested regions that might be imprinted, further analyses using variance-components method failed to obtain significant signals of imprinting. The second data set was Problem 1 of GAW15, which included single-nucleotide polymorphism genotypes and gene expression data for Centre d'Etude du Polymorphisme Humain pedigrees. A previous genome-wide linkage scan identified loci that may be regulators of gene expression: our genome-wide joint linkage and imprinting scan using a variance-components approach found significant signals for linkage.

**Conclusion:**

Our linkage scan results suggest that imprinted genes are unlikely to be involved in susceptibility to rheumatoid arthritis. However, for expression level of *TGFBR3 *gene, we found a point-wise *p*-value of 0.03 for imprinting, but increase in the LOD score did not meet the required threshold to reliably identify imprinting as the correct mode of inheritance in genome-wide linkage scans.

## Background

Genomic imprinting is a phenomenon in which the expression level of a gene is determined by the parental origin of the chromosome on which it resides. In maternal imprinting, genes that are on the paternally inherited chromosome are expressed while their counterparts on the maternally inherited chromosomes are silenced. Several genes that affect development in mammals are known to be imprinted, and imprinted genes have been implicated in several human disorders. Currently, only 41 transcriptional units have been identified as imprinted in humans [1]. However, because Luedi et al. [2] recently predicted that about 600 genes are imprinted in mice, we suspect that there are many undiscovered imprinted genes in the human genome. Incorporating information on imprinting may improve the power to detect linkage if the locus of interest is in fact imprinted. In 2002, Shete and Amos [3] presented a framework allowing parent-of-origin information in linkage analyses of quantitative traits.

In our analyses of North American Rheumatoid Arthritis Consortium (NARAC) data, we used measurements of anti-cyclic citrullinated peptide (CCP) antibody as quantitative trait values. Because these quantitative phenotypes are part of the diagnosis of rheumatoid arthritis, the variation in these phenotypes should be due to the same underlying variation in genetic factors as in rheumatoid arthritis. In the analyses of gene expression data (Problem 1 of GAW15), we used single-nucleotide polymorphism (SNP) data of 14 Centre d'Etude du Polymorphisme Humain (CEPH) Utah pedigrees. The expression levels of 60 genes (Additional file 1) in lymphoblastoid cells were used as quantitative traits. These 60 genes are among the 3554 that have shown greater variation between individuals than within the same individual [4]. Fifty-nine of these genes were selected because they showed linkage signals for *trans*-acting regulator loci in a linkage scan [5]. In addition to these 59 genes, asparagine-linked glycosylation 6 homolog (*ALG6*) was also included because a *trans*-acting regulator of *ALG6 *was mapped to chromosome 19 in a previous genome-wide linkage scan [6]. The distributions of these expression levels were either approximately normal or were transformed using Box-Cox transformation, which is a requirement of the maximum-likelihood method that we used to estimate parameters. In our genome-wide joint linkage and imprinting scan, likelihood ratio tests were carried out using two models for each trait, one with imprinting and one without. In the case of *ALG6*, we compared results obtained using our method with the previous linkage scan based on Haseman-Elston sib-pair based regression approach [6].

## Methods

### Parametric imprinting method for rheumatoid arthritis

We used previously developed parametric linkage and imprinting methods [7,8]. Briefly, at the trait locus, let D be the deleterious allele and N the normal allele. To account for imprinting we specify four penetrance values: P(y|DD), P(y|DN), P(y|ND), and P(y|NN). Here, without loss of generality, we assume that the first allele is derived from the father and the second allele is derived from the mother. The difference in penetrance values P(y|DN) and P(y|ND) indicates the presence of imprinting. In this study, we considered a difference greater than 0.9 to be suggestive of imprinting. We conducted multipoint MOD score analysis with GENEHUNTER-MODSCORE [9].

### Model-free imprinting method for quantitative traits for rheumatoid arthritis and expression data

For gene expression data of CEPH pedigrees, we conducted a genome-wide linkage scan using model-free methods as described previously [10]. For NARAC data, only regions showing imprinting in parametric MOD score linkage analyses were further evaluated. These model-free methods are variance-components based approaches that allow for genetic imprinting. We used Simwalk [11] to obtain estimates of 15 detailed states of identity-by-descent (IBD) sharing at each marker. We then calculated parent-specific IBD sharing between pairs of individuals in a family. The likelihood-ratio test was conducted using a modified version of ACT, which allows extra parameters to model imprinting.

### Rheumatoid arthritis data

To identify imprinted genes that affect the risk for rheumatoid arthritis, we used data from 659 Caucasian and 29 Hispanic families provided by NARAC and the Canadian group [12]. In the Caucasian families, there were a total of 1577 affected persons, of whom a total of 1476 individuals had genotypes available. In the Hispanic families, we had a total of 67 persons affected, with genotypes available for 63. We analyzed both qualitative data (affected or unaffected) and quantitative data (the anti-CCP antibody).

### Gene expression data

We analyzed SNP data of 14 CEPH Utah pedigrees. A total of 2819 autosomal SNP markers were typed in 194 individuals by the SNP Consortium [13]. In our analyses, we used gene expression levels in lymphoblastoid cells as quantitative traits. We selected the expression levels of 59 genes that had linkage signals for *trans*-acting regulators [5] and *ALG6 *because a *trans*-acting regulator of *ALG6 *was mapped to chromosome 19 in a previous genome-wide linkage scan (Additional file 1). Expression data and marker genotype were available for all parents and an average of eight offspring per sibship.

## Results and discussion

We used two models in genome scans, one allowing for imprinting and one without. We obtained multipoint LOD scores for 22 autosomal chromosomes under both models. The highest multipoint LOD scores for each gene were listed in Additional file 1. For testing linkage without imprinting, the likelihood ratio test (2 × Log_e_(10) × LOD_Reg_) asymptotically follows a half-and-half mixture of χ^2 ^random variable with 1 and 0 degrees of freedom. A LOD score of 3.0 is considered significant for genome-wide linkage scan, which gives an asymptotic *p*-value of 0.0001. For joint testing of linkage and imprinting, the likelihood ratio test (2 × Log_e_(10) × LOD_Imp_) asymptotically follows a mixture of χ^2 ^distributions with 0, 1, and 2 degrees of freedom in the proportions of 1/4, 1/2, and 1/4. Therefore, to achieve an asymptotic *p*-value of 0.0001, a LOD_Imp _score of 3.4 is needed. Under the no imprinting variance-components model, expression levels of three genes had LOD scores greater than 3.0. These LOD scores were 4.08 on chromosome 2, 4.15 on chromosome 2, and 3.26 on chromosome 20, for transforming growth factor beta receptor III (*TGFBR3*), solute carrier family 25 member 32 (*SLC25A32*), and early growth response 2 (*EGR2*), respectively. Under the model that allows for imprinting, we obtained LOD scores of 4.86 on chromosome 2, 4.29 on chromosome 2, and 3.79 on chromosome 20, for *TGFBR3*, *SLC25A32*, and *EGR2*, respectively. No additional linkage signals were observed under the imprinting model. It is important to note that the maximum LOD scores either obtained by allowing for imprinting or obtained without allowing for imprinting were at the same or nearby markers. We used a statistic Δ = 2 × Log_e_(10) × (LOD_Imp_-LOD_Reg_) to obtain *p*-values for the test of imprinting where LOD_Imp _and LOD_Reg _are LOD scores using imprinting model and no imprinting model, respectively. The Δ statistic is a likelihood ratio test that is asymptotically a mixture of 0 and χ^2^, with one degree of freedom in the proportions of 1/2 and 1/2 under the null hypothesis. For *TGFBR3*, the difference between LOD_Imp _and LOD_reg _was 0.78 at marker rs117261, where maximum LOD score was observed under the imprinting model. Based on Δ statistic, *TGFBR3 *showed *p*-value of 0.03 for imprinting. The estimated values of variance components suggested maternal expression. Greenberg et al. [14] proposed that a difference of greater than 1.5 between LOD scores is needed to reliably identify the correct mode of inheritance in genome-wide linkage scans.

We conducted simulation studies to evaluate power to detect linkage of an imprinted locus using the 14 CEPH families provided by GAW. We simulated a quantitative trait that had 20% heritability and was tightly linked to a marker locus (theta = 0) with eight equally frequent alleles (to simulate the nearly complete information about segregation provided by the dense SNP scan). The trait locus had two alleles (allele frequency of A was 0.3) and was maternally imprinted. The means of trait values were set at 0.68 for genotypes AA (first allele being the paternally inherited allele) and Aa, and -0.68 for aA and aa. We also simulated a separate locus unlinked to the marker to represent the polygenic component of genetic variance. The unlinked locus had two equally frequent alleles (B and b) with means of 1.0 for genotype BB, 0.0 for genotypes Bb and bB, and -1.0 for genotype bb. The total residual variance was 1.0. Under these conditions, we observed an average LOD score of 3.38 from 100 replicates which suggest that an imprinted disease locus with 20% heritability can be detected using these families.

For *ALG6*, the highest LOD score of 2.18 was obtained at rs1019937 on chromosome 19 when imprinting was not allowed. This LOD score is suggestive for linkage but not significant in a genome-wide scan. Our result is different from previous reports by Morley et al. [6], who identified strong evidence of linkage for a *trans*-acting regulator of *ALG6 *on chromosome 19 (*p*-value < 10^-9^). Furthermore, when we included imprinting in our linkage analyses, we obtained a LOD score of 2.34 at the same marker location. The difference in LOD scores, with imprinting and without imprinting, is not significant for concluding that imprinting is a mode of inheritance.

Linkage analyses of NARAC data was conducted in two stages. We first performed multipoint MOD score analysis over the entire genome with GENEHUNTER-MODSCORE [9]. We then selected SNPs that showed a difference larger than 0.9 in estimated values of P(y|DN) and P(y|ND) and a corresponding MOD score that was larger than 1.0. Then, we used a model-free variance components based imprinting method using anti-CCP antibody as a quantitative trait at these select loci. Our MOD score analyses did not reveal any imprinted regions in Caucasian families. However, two regions showed significant imprinting in Hispanic families (chromosome 1, 114.48–120.20 Mb region in Build 36 and chromosome 10, 75.34–80.60 Mb region in Build 36). We obtained MOD scores of 2.32 on chromosome 1 and 2.05 on chromosome 10. At both locations, the estimated values for P(y|DN) and P(y|ND) were 0.00 and 1.00, respectively. We performed variance components-based model-free joint linkage and imprinting analyses. Theses analyses did not show significance of imprinting in these two regions (*p*-value > 0.5). Our results suggested that imprinted genes are unlikely to be involved in susceptibility to rheumatoid arthritis.

In our analyses of NARAC data, we observed differences between Caucasian and Hispanic families that were pronounced in the distributions of age of onset, anti-CCP antibody, and other variables. These differences may be caused by the distinct genetic backgrounds of the two groups.

We identified three regulatory loci of gene expression in our genome scans. We found a point-wise *p*-value of 0.03 for imprinting for the *TGFBR3 *gene, which is of interest, but the increase in the LOD score did not meet the threshold of 1.5 to reliably identify imprinting as the correct mode of inheritance in genome-wide linkage scans. However, our search is exploratory and far from exhaustive. We only included genes that had shown evidence of linkage in previous studies. With proper transformation, expression levels of other genes can be analyzed in a similar fashion. Whether some other regulators of gene expression may be imprinted is still a question that deserves further investigation. Our simulation study indicates that our method has ample power to detect linkage under imprinting using pedigrees similar in size to the CEPH families.

Our analyses of *ALG6 *expression data yielded results less significant than those previously published, perhaps because of differences in the raw data (SNP density) and statistical methods that were used. Previous results were obtained using a modified version of Haseman and Elston method [15], which is less sensitive to the normality of distribution of quantitative traits but excludes quantitative phenotypes from parents and grandparents. We did notice differences in mean levels across generations and this was not accounted for in our analysis. It is possible that a true linkage might be overlooked or reduced in signal, due to these mean shifts across generations. To investigate this possibility, we re-analyzed the CEPH pedigrees for *ALG6*, excluding parents and grandparents. We obtained much higher maximum LOD scores on chromosome 19 (3.61 without imprinting and 3.76 with imprinting), which are significant for linkage, but difference between the two were not suggestive for imprinting.

## Conclusion

Our linkage scan results suggest that imprinted genes are unlikely to be involved in susceptibility to rheumatoid arthritis. However, for *TGFBR*3 gene, we found a point-wise *p*-value of 0.03 for imprinting, but the increase in the LOD score did not meet the required threshold to reliably identify imprinting as the correct mode of inheritance in genome-wide linkage scans.

## Competing interests

The author(s) declare that they have no competing interests.

## Authors' contributions

SS and XZ participated in project conception, and drafted the manuscript. XZ performed nonparametric linkage analyses of NARAC and expression data. WC performed MOD score analyses of NARAC data. RY suggested changes in the analyses. YL and RY participated in data reformatting and management. MDS, C-CW, and CIA contributed in discussions of results and revision of the manuscript.

## Supplementary Material

Additional file 1 Genome scan of gene expression data using MULTIC-IMPRINTING. Click here for file

## References

[B1] Morison IM, Ramsay JP, Spencer HG (2005). A census of mammalian imprinting. Trends Genet.

[B2] Luedi PP, Hartemink AJ, Jirtle RL (2005). Genome-wide prediction of imprinted murine genes. Genome Res.

[B3] Shete S, Amos CI (2002). Testing for genetic linkage in families by a variance-components approach in the presence of genomic imprinting. Am J Hum Genet.

[B4] Cheung VG, Conlin LK, Weber TM, Arcaro M, Jen KY, Morley M, Spielman RS (2003). Natural variation in human gene expression assessed in lymphoblastoid cells. Nat Genet.

[B5] Yu R, DeHoff K, Amos CI, Shete S (2007). Seeking gene relationship from gene expression data using support vector machine regression. BMC Proc.

[B6] Morley M, Molony CM, Weber TM, Devlin JL, Ewens KG, Spielman RS, Cheung VG (2004). Genetic analysis of genome-wide variation in human gene expression. Nature.

[B7] Strauch K, Fimmers R, Kurz T, Deichmann KA, Wienker TF, Baur MP (2000). Parametric and nonparametric multipoint linkage analysis with imprinting and two-locus-trait models: application to mite sensitization. Am J Hum Genet.

[B8] Shete S, Zhou X (2005). Parametric approach to genomic imprinting analysis with applications to Angelman's syndrome. Hum Hered.

[B9] Strauch K, Furst R, Ruschendorf F, Windemuth C, Dietter J, Flaquer A, Baur MP, Wienker TF (2005). Linkage analysis of alcohol dependence using MOD scores. BMC Genet.

[B10] Shete S, Zhou X, Amos CI (2003). Genomic imprinting and linkage test for quantitative-trait loci in extended pedigrees. Am J Hum Genet.

[B11] Weeks DE, Sobel E, O'Connell JR, Lange K (1995). Computer programs for multilocus haplotyping of general pedigrees. Am J Hum Genet.

[B12] Amos CI, Chen WV, Lee A, Li W, Kern M, Lundsten R, Batliwalla F, Wener M, Remmers E, Kastner DA, Criswell LA, Seldin MF, Gregersen PK (2006). High-density SNP analysis of 642 Caucasian families with rheumatoid arthritis identifies two new linkage regions on 11p12 and 2q33. Genes Immun.

[B13] Matise TC, Sachidanandam R, Clark AG, Kruglyak L, Wijsman E, Kakol J, Buyske S, Chui B, Cohen P, de Toma C, Ehm M, Glanowski S, He C, Heil J, Markianos K, McMullen I, Pericak-Vance MA, Silberglet A, Stein L, Wagner M, Wilson AF, Winick JD, Winn-Deen ES, Yamashiro CT, Cann HM, Lai E, Holden AL (2003). A 3.9-centimorgan-resolution human single-nucleotide polymorphism linkage map and screening set. Am J Hum Genet.

[B14] Greenberg DA, Berger B (1994). Using lod-score differences to determine mode of inheritance – a simple, robust method even in the presence of heterogeneity and reduced penetrance. Am J Hum Genet.

[B15] Shete S, Jacobs KB, Elston RC (2003). Adding further power to the Haseman and Elston method for detecting linkage in larger sibships: weighting sums and differences. Hum Hered.

