# Supplementary material for: Joint linkage and imprinting analyses of GAW15 rheumatoid arthritis and gene expression data
Source: BMC Proc. 2007 Dec 18;1(Suppl 1):S53. doi: 10.1186/1753-6561-1-s1-s53 (PMC2367552; doi:10.1186/1753-6561-1-s1-s53)
Supplement: Additional file 1 — Genome scan of gene expression data using MULTIC-IMPRINTING. [file 1753-6561-1-S1-S53-S1.doc]

## Genome scan of gene expression data using MULTIC-IMPRINTING

| Gene | Location | LODreg | LODimp |
| --- | --- | --- | --- |
| ***TGFBR3*^a^** | **1p33-p32** | **4.08 (chr 2)** | **4.86 (chr 2)** |
| ***SLC25A32*** | **8q22.3** | **4.15 (chr 2)** | **4.29 (chr 2)** |
| ***EGR2*** | **10q21.1** | **3.26 (chr 20)** | **3.79 (chr 20)** |
| *DPH2* | 1p34 | 1.66 (chr 2) | 2.92 (chr 2) |
| *SPINT1* | 15q15.1 | 2.36 (chr 8) | 2.91 (chr 8) |
| *CYB561D2* | 3p21.3 | 1.73 (chr 11) | 2.91 (chr 11) |
| *ITGB7* | 12q13.13 | 2.89 (chr 9) | 2.89 (chr 9) |
| *NFIB* | 9p24.1 | 2.84 (chr 20) | 2.84 (chr 20) |
| *ARL2BP* | 16q13 | 2.83 (chr 14) | 2.83 (chr 14) |
| *SGPP1* | 14q23.2 | 2.81 (chr 2) | 2.81 (chr 2) |
| *CHST11* | 12q | 2.78 (chr 16) | 2.78 (chr 16) |
| *TNFRSF10B* | 8p22-p21 | 2.06 (chr 14) | 2.78 (chr 14) |
| *PCYT1A* | 3q29 | 2.76 (chr 13) | 2.76 (chr 13) |
| *FIGF* | Xp22.31 | 2.70 (chr 11) | 2.70 (chr 11) |
| *RAB31* | 18p11.3 | 1.83 (chr 12) | 2.64 (chr 12) |
| *RAP80* | 5q35.2 | 2.63 (chr 14) | 2.63 (chr 14) |
| *BUB1B* | 15q15 | 2.61 (chr 11) | 2.61 (chr 11) |
| *ARHGAP6* | Xp22.3 | 1.76 (chr 14) | 2.59 (chr 14) |
| *APS* | 7q22 | 1.96 (chr 11) | 2.53 (chr 11) |
| *ALDH9A1* | 1q23.1 | 2.46 (chr 20) | 2.48 (chr 20) |
| *CCL4* | 17q12 | 2.45 (chr 12) | 2.45 (chr 12) |
| *NCR3* | 6p21.3 | 2.45 (chr 1) | 2.45 (chr 1) |
| *ALG6* | 1p31 | 2.18 (chr 19) | 2.34 (chr 19) |
| *LGALS9* | 17q11.2 | 2.31 (chr 14) | 2.31 (chr 14) |
| *PDIA3* | 15q15 | 2.24 (chr 14) | 2.24 (chr 14) |
| *VBP1* | Xq28 | 2.23 (chr 14) | 2.23 (chr 14) |
| *NFYA* | 6p21.3 | 1.80 (chr 20) | 2.20 (chr 20) |
| *EIF1AY* | Yq11.222 | 1.47 (chr 19) | 2.12 (chr 19) |
| *CDC42EP4* | 17q24-q25 | 2.11 (chr 13) | 2.11 (chr 13) |
| *UTX* | Xp11.2 | 2.11 (chr 10) | 2.11 (chr 10) |
| *INPP5A* | 10q26.3 | 2.10 (chr 14) | 2.10 (chr 14) |
| *LIN7C* | 11p14 | 1.64 (chr 14) | 2.08 (chr 14) |
| *MIR16* | 16p12-p11.2 | 2.05 (chr 14) | 2.05 (chr 14) |
| *ACOT9* | Xp22.11 | 2.02 (chr 9) | 2.02 (chr 9) |
| *YAF2* | 12q12 | 2.02 (chr 9) | 2.02 (chr 9) |
| *DDX5* | 17q21 | 1.98 (chr 8) | 1.98 (chr 8) |
| *PIM1* | 6p21.2 | 1.95 (chr 9) | 1.95 (chr 9) |
| *HRB* | 2q36.3 | 1.89 (chr 14) | 1.89 (chr 14) |
| *SNAPC4* | 9q34.3 | 1.30 (chr 16) | 1.88 (chr 16) |
| *SEC61B* | 9q22.32-q31.3 | 1.87 (chr 3) | 1.87 (chr 3) |
| *PHTF1* | 1p13 | 1.84 (chr 21) | 1.85 (chr 21) |
| *ACP6* | 1q21 | 1.28 (chr 20) | 1.78 (chr 20) |
| *YWHAQ* | 2p25.1 | 1.77 (chr 19) | 1.77 (chr 19) |
| *HIRA* | 22q11.2|22q11.21 | 1.66 (chr 7) | 1.66 (chr 7) |
| *RERE* | 1p36.1-p36.2 | 1.57 (chr 11) | 1.59 (chr 11) |
| *NUDC* | 1p35-p34 | 1.44 (chr 4) | 1.56 (chr 4) |
| *SCML1* | Xp22.2-p22.1 | 1.09 (chr 7) | 1.55 (chr 7) |
| *FOXG1B* | 14q12-q13 | 1.54 (chr 22) | 1.54 (chr 22) |
| *KIF23* | 15q23 | 1.54 (chr 13) | 1.54 (chr 13) |
| *CDH1* | 16q22.1 | 1.52 (chr 17) | 1.52 (chr 17) |
| *OAS3* | 12q24.2 | 1.46 (chr 3) | 1.46 (chr 3) |
| *LRRC32* | 11q13.5-q14 | 1.36 (chr 7) | 1.41 (chr 7) |
| *ETNK1* | 12p12.1 | 1.29 (chr 16) | 1.38 (chr 16) |
| *ACSM3* | 16p13.11 | 1.33 (chr 12) | 1.33 (chr 12) |
| *CD9* | 12p13.3 | 1.16 (chr 2) | 1.16 (chr 2) |
| *ZNF224* | 19q13.2 | 1.06 (chr 18) | 1.06 (chr 18) |
| *EFEMP2* | 11q13 | 0.96 (chr 3) | 0.96 (chr 3) |
| *SLC22A5* | 5q31 | 0.68 (chr 11) | 0.68 (chr 11) |
| *GCA* | 2q24.2 | 0.41 (chr 12) | 0.50 (chr 12) |
| *THRAP1* | 17q22-q23 | 0.34 (chr 3) | 0.43 (chr 3) |

**^a^Highest LOD scores under the imprinting model are listed first. Significant LOD scores are in bold font.**
